# Positionspapier: Open-source-Technologie in der Behandlung von Menschen mit Diabetes mellitus – eine österreichische Perspektive: Technologieausschuss der Österreichischen Diabetes Gesellschaft

**DOI:** 10.1007/s00508-024-02400-x

**Published:** 2024-08-28

**Authors:** Antonia-Therese Kietaibl, Ingrid Schütz-Fuhrmann, Latife Bozkurt, Lisa Frühwald, Birgit Rami-Merhar, Elke Fröhlich-Reiterer, Sabine E. Hofer, Martin Tauschmann, Michael Resl, Thomas Hörtenhuber, Lars Stechemesser, Yvonne Winhofer, Michaela Riedl, Sandra Zlamal-Fortunat, Marlies Eichner, Harald Stingl, Christian Schelkshorn, Raimund Weitgasser, Gersina Rega-Kaun, Gerd Köhler, Julia K. Mader

**Affiliations:** 1 5. Medizinische Abteilung für Endokrinologie, Rheumatologie und Akutgeriatrie, Klinik Ottakring, Wien, Österreich; 2 Verein zur Förderung der wissenschaftlichen Forschung am Wilhelminenspital, Wien, Österreich; 3 3. Medizinische Abteilung mit Stoffwechselerkrankungen und Nephrologie, Karl Landsteiner Institut für Endokrinologie und Stoffwechselerkrankungen, Klinik Hietzing, Wien, Österreich; 4 Universitätsklinik für Kinder- und Jugendheilkunde, Abteilung für Pädiatrische Pulmologie, Allergologie und Endokrinologie, Medizinische Universität Wien, Wien, Österreich; 5 Universitätsklinik für Kinder- und Jugendheilkunde, Abteilung für Allgemeine Pädiatrie, Medizinische Universität Graz, Graz, Österreich; 6 Department für Pädiatrie 1, Medizinische Universität Innsbruck, Innsbruck, Österreich; 7 Klinische Abteilung für Endokrinologie und Diabetologie, Universitätsklinik für Innere Medizin, Medizinische Universität Graz, Graz, Österreich; 8 Abteilung für Innere Medizin I, Konventhospital der Barmherzigen Brüder Linz, Linz, Österreich; 9 Klinische Abteilung für Endokrinologie und Stoffwechsel, Universitätsklinik für Innere Medizin III, Medizinische Universität Wien, Wien, Österreich; 10 Abteilung für Innere Medizin und Gastroenterologie, Hepatologie, Endokrinologie, Rheumatologie und Nephrologie, Klinikum Klagenfurt am Wörthersee, Klagenfurt, Österreich; 11 Klinische Abteilung für Endokrinologie und Diabetologie, Medizinische Universität Graz, Graz, Österreich; 12 Rehabilitation für Stoffwechselerkrankungen Aflenz, Aflenz, Österreich; 13 Universitätsklinik für Kinder- und Jugendheilkunde, Kepler Universitätsklinikum, Linz, Österreich; 14 Universitätsklinik für Innere Medizin I, Paracelsus Medizinische Privatuniversität, Salzburg, Österreich; 15 Kompetenzzentrum Diabetes, Abteilung für Innere Medizin, Privatklinik Wehrle-Diakonissen, Salzburg, Österreich; 16 Universitätsklinik für Innere Medizin I, Paracelsus Medizinische Privatuniversität, Salzburg, Österreich; 17 Abteilung Innere Medizin, Landesklinikum Baden, Baden, Österreich; 18 1. Medizinische Abteilung, Landesklinikum Stockerau, Stockerau, Österreich

**Keywords:** Diabetes mellitus, Diabetestechnologie, Automatisierte Insulinabgabesysteme, Open-source-Technologie, Do-it-Yourself-Pankreassysteme, Diabetes mellitus, Diabetes technology, Automated insulin delivery systems, Open-source technology, Do it yourself pancreas system

## Abstract

Menschen mit Diabetes mellitus können im alltäglichen Management durch Diabetestechnologie mittels automatisierter Insulinabgabesysteme (AID-Systeme) unterstützt werden und dadurch das Hypoglykämierisiko reduzieren und die glykämische Kontrolle sowie die Lebensqualität verbessern. Aufgrund von unterschiedlichsten Barrieren in der AID-Verfügbarkeit hat sich international die Nutzung von Open-source-AID-Systemen entwickelt. Diese Technologien bieten eine notwendige Alternative zu kommerziellen Produkten, insbesondere, wenn zugelassene Systeme unzugänglich oder unzureichend auf die spezifischen Bedürfnisse der Anwendenden angepasst sind. Open-source-Technologie zeichnet sich durch global freie Verfügbarkeit von Codes im Internet aus, durchläuft kein offizielles Zulassungsverfahren, und die Verwendung erfolgt daher auf eigene Verantwortung. In der klinischen Praxis führen fehlende Expertise zu den unterschiedlichen Systemen und Bedenken vor juristischen Konsequenzen zu Konfliktsituationen für Behandler:innen und mitunter zur Ablehnung in der Betreuung von Menschen mit Diabetes mellitus, die Open-source-Technologie nutzen möchten. Im vorliegenden Positionspapier sollen eine Übersicht zu vorhandener Evidenz sowie praktische Orientierungshilfen für medizinisches Fachpersonal geboten werden, um Unsicherheiten und Barrieren zu minimieren. Menschen mit Diabetes mellitus müssen – unabhängig von der von ihnen gewählten Diabetestechnologie – weiterhin in Schulung, Umgang und Management ihrer Erkrankung unterstützt werden, auch wenn sie sich für die Verwendung eines Open-source-Systems entschieden haben. Medizinische Kontrollen der metabolischen Einstellung, akuter und chronischer Komplikationen sowie das Screening auf assoziierte Erkrankungen sind unabhängig vom gewählten AID-System notwendig und sollen durch multidisziplinäre Teams mit entsprechender Expertise erfolgen.

## Abstract (en)

People living with diabetes mellitus can be supported in the daily management by diabetes technology with automated insulin delivery (AID) systems to reduce the risk of hypoglycemia and improve glycemic control as well as the quality of life. Due to barriers in the availability of AID-systems, the use and development of open-source AID-systems have internationally increased. This technology provides a necessary alternative to commercially available products, especially when approved systems are inaccessible or insufficiently adapted to the specific needs of the users. Open-source technology is characterized by worldwide free availability of codes on the internet, is not officially approved and therefore the use is on the individual’s own responsibility. In the clinical practice a lack of expertise with open-source AID technology and concerns about legal consequences, lead to conflict situations for health-care professionals (HCP), sometimes resulting in the refusal of care of people living with diabetes mellitus. This position paper provides an overview of the available evidence and practical guidance for HCP to minimize uncertainties and barriers. People living with diabetes mellitus must continue to be supported in education and diabetes management, independent of the chosen diabetes technology including open-source technology. Check-ups of the metabolic control, acute and chronic complications and screening for diabetes-related diseases are necessary and should be regularly carried out, regardless of the chosen AID-system and by a multidisciplinary team with appropriate expertise.

Menschen mit Diabetes mellitus Typ 1 und anderen Diabetesformen mit Insulinmangel leben mit einer chronischen Stoffwechselerkrankung, die leitliniengerecht eine komplexe Insulintherapie notwendig macht. Hierbei gilt es, tägliche Herausforderungen des Alltags bestmöglich zu meistern, um therapeutische Ziele laut Leitlinien (HbA_1c_ < 7 % bzw. < 53 mmol/mol; < 6,5 % bzw. < 47,5 mmol/mol ohne Hypoglykämien bzw. Time in Range [TIR] > 70%) zu erreichen. Die glykämische Kontrolle ist entscheidend, um diabetesassoziierte Komplikationen zu reduzieren [1–4]. Automatisierte Insulinabgabesysteme (AID-Systeme) konnten in zahlreichen Studien demonstrieren, dass es durch ihren Einsatz zu einer Reduktion des Hypoglykämierisikos und einer Verbesserung der glykämischen Kontrolle sowie der Lebensqualität kommt [5–9]. Bei AID-Systemen, auch als Hybrid-Closed-Loop-Systeme bezeichnet, reguliert ein Algorithmus die Insulinzufuhr automatisiert über die Pumpe unter Berücksichtigung der aktuellen und vergangenen Glukoseverläufe, generiert durch kontinuierliche Glukosemessung (CGM; Abb. 1; [10, 11]). Hybrid-Closed-Loop beschreibt hierbei die Tatsache, dass in den aktuell verfügbaren AID-Systemen Kohlenhydrateingaben vorgenommen und manuelle Bolusabgaben über die Insulinpumpe getätigt werden müssen [12]. Trotz technologischer Fortschritte werden die vorgegebenen Therapieziele international weiterhin nicht flächendeckend erreicht, und der weltweite Technologieeinsatz ist regional äußerst unterschiedlich. Ungleichheiten im Zugang (städtische vs. ländliche Gebiete, Betreuung in Spezialambulanzen), in der internationalen Erstattung sowie der Verfügbarkeit kommerziell erhältlicher AID-Systeme sind dafür verantwortlich.

**Abb. 1:**
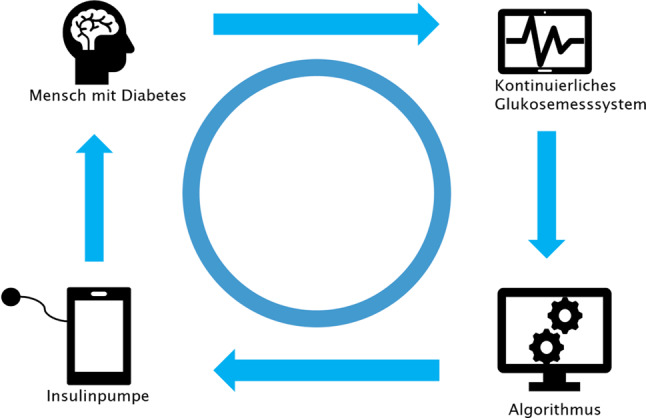
Automatisiertes Insulinabgabesystem (AID-System). (Mod. nach [11])

Darüber hinaus beklagen Menschen mit Diabetes mellitus die fehlende freie Entscheidung hinsichtlich Zusammensetzung der Komponenten eines AID-Systems, da die Interoperabilität zwischen unterschiedlichen Glukosesensoren und Insulinpumpenmodellen herstellerseitig nicht gefördert wird [13–18]. Der Begriff Interoperabilität bezeichnet hierbei die Fähigkeit einzelner Komponenten eines AID-Systems, miteinander zu kommunizieren, indem Informationsaustausch und -nutzung uneingeschränkt und herstellerunabhängig möglich sind [17]. Open-source-AID-Systeme (Open-source-AID) wurde von technologieaffinen Personen wie Angehörigen von oder Menschen mit Diabetes mellitus selbst entwickelt, um AID auch abseits von kommerziellen Produkten nutzen zu können. Der Terminus „Open-source“ (oder Do-it-Yourself) bedeutet, dass die verwendeten Technologien in einer Gemeinschaft von Menschen bzw. Angehörigen von Menschen mit Diabetes mellitus (#WeAreNotWaiting-Community) entwickelt wurden, welche für die Nutzung frei im Internet abrufbar zur Verfügung stehen, jedoch – zum Großteil – keine offiziellen Zulassungsprozesse durchlaufen mussten [19, 20]. Die intensive Beschäftigung mit den verwendeten Algorithmen und Einstellungen führt in den meisten Fällen zu einer hohen Expertise aufseiten der Menschen mit Diabetes mellitus bzw. deren Betreuungspersonen, während diabetologisch tätige Ärzt:innen teilweise wenig konkrete Erfahrung mit diesen Systemen haben. Letzteres führt im klinischen Alltag zu einer Konfliktsituation für das medizinische Fachpersonal, wobei fehlendes Wissen zu Open-source-Technologien und Bedenken vor medizinisch-juristischen Konsequenzen als besondere Herausforderungen bestehen [21–25]. Im vorliegenden Positionspapier umfasst der Begriff „Betreuungspersonen“ alle Angehörigen von Menschen mit Diabetes mellitus, die als Familien, Eltern, Erziehungsberechtigte oder gesetzliche Vertreter:innen in das Diabetesmanagement involviert sind.

## Ziele des Positionspapiers

Im vorliegenden Positionspapier soll die klinische Herausforderung im Umgang mit Open-source-AID erörtert werden, und eine Übersicht vorhandener Evidenz sowie praktische Orientierungshilfe für medizinisches Fachpersonal sollen geboten werden, um Unsicherheiten und Barrieren zu minimieren. Bedenken aufseiten des medizinischen Fachpersonals durch die Betreuung von Menschen mit Diabetes mellitus mit Open-source-Technologie sollen durch Aufklärung reduziert werden. Als relevant erachtet der Technologieausschuss der Österreichischen Diabetes Gesellschaft die Gefahr, dass Menschen mit Diabetes mellitus, die sich für die Nutzung eines Open-source-AID entscheiden und Ablehnung beim medizinischen Personal erfahren, keine regelmäßigen diabetologischen Kontrollen mehr wahrnehmen und dadurch Nachteile in der Betreuungssituation – insbesondere hinsichtlich Risikofaktormanagements und Komorbiditäten – zu erwarten sind. Menschen mit Diabetes mellitus sollen – unabhängig von der von ihnen gewählten Diabetestechnologie – weiterhin im Umgang und Management unterstützt werden, auch wenn sie sich für die Verwendung eines Open-source-AID entschieden haben. Die Entscheidung für oder gegen die Verwendung eines Open-source Systems können Menschen mit Diabetes mellitus nur nach entsprechender Aufklärung über Vor- und Nachteile dieser Technologie treffen. Diese Information zu teilen ist die Aufgabe des informierten betreuenden Diabetesteams. Das Positionspapier bezieht sich auf in der Literatur verfügbares Expert:innenwissen, auf die klinische Erfahrung der beteiligten Autor:innen sowie vorhandene Evidenz. Im vorliegenden Papier wird kein allgemeiner Überblick vorhandener Diabetestechnologien gegeben, da dies bereits Inhalt anderer Leitlinien ist [1, 5].

## Open-source-Technologie im Diabetesmanagement – Entstehung und Evidenz

Bereits Anfang der 2000er-Jahre fand die sensorunterstützte Insulinpumpentherapie Einzug in die Praxis. Menschen mit Diabetes mellitus sind und waren jedoch abhängig von der nationalen Verfügbarkeit und Erstattungssituation. Aus dem Wunsch einer individualisierten Therapiesteuerung mittels modifizierter CGM-Alarme, veränderbarer Glukosezielwerte, Datengenerierung in der Cloud und dem Traum eines künstlichen Pankreas (englisch: „artificial pancreas system“ [APS]), entstand die #WeAreNotWaiting-Bewegung [19, 26, 27].

Die Do-it-Yourself-Community zeichnet sich durch globalen Support über soziale Netzwerke, regionale Vernetzung, Expert:innen abseits von ärztlichem Personal und freie Verfügbarkeit der notwendigen Codes zum selbstständigen Programmieren der Systeme aus. Es gab bereits 2013/14 erste Fallberichte zur Nutzung eines Do-it-Yourself-Pankreassystems (DIYPS), und ab 2015 kam es zur steigenden Verwendung dieser DIYPS, bevor 2016 das erste kommerzielle Hybrid-Closed-Loop-System die offizielle Zulassung erreichte. Schätzungen zufolge nutzen weltweit ca. 10.000 Menschen Open-source-AID [21, 26, 28, 29]. Zahlen für den österreichischen Einsatz von Open-source-AID können nur schätzungsweise anhand von lokalen Gruppen auf sozialen Netzwerken angegeben werden. Diese belaufen sich im Erwachsenenbereich auf ca. 95 Menschen mit Diabetes mellitus und aktiver Open-source-AID-Nutzung, wobei AndroidAPS häufiger als iAPS und IOS-Loop genutzt werden dürfte. In der pädiatrischen Diabetologie dürfte die Nutzung bei unter 5 % liegen. Die tatsächlich zu erwartende Anzahl an Nutzer:innen ist vermutlich höher anzusetzen, und es ist von einer ständig wachsenden Gemeinschaft auszugehen. Valide verwertbare Daten fehlen hierzu in Österreich.

Wie einleitend beschrieben, ist die nationale Verfügbarkeit kommerzieller AID-Systeme sehr unterschiedlich. Trotz rasanter technologischer Fortschritte sind in Österreich aktuell (Stand Mai 2024) lediglich 2 zugelassene AID-Systeme erhältlich, davon weiterhin trotz generellen Vorhandenseins und CE-Zertifizierung keine schlauchlose AID-Option. Auch die Auswahlmöglichkeit des CGM-Systems sowie eines ggf. notwendigen und kompatiblen Smartphones ist durch die eingeschränkte Kompatibilität/Interoperabilität zwischen unterschiedlichen Herstellerfirmen nicht frei wählbar. Dieser Tatsache möchten die Open-source-Technologien Rechnung tragen [17].

Unterschiedliche Fachgesellschaften bzw. Diabeteszentren in Australien, dem Vereinigten Königreich sowie Dänemark haben aufgrund steigender Nutzung von Open-source-AID seit 2019/2020 Positionspapiere veröffentlicht, wobei im Kern das Risiko bei Nutzung von nicht zugelassenen Systemen hervorgehoben wurde [30–33]. Eine aus der Open-source-Initiative hervorgegangene App namens Tidepool-Loop, die entwickelt wurde, um das Problem der mangelnden Kompatibilität zwischen verschiedenen CGM- und Insulinpumpensystemen zu lösen, hat inzwischen die Zulassung der Food and Drug Administration (FDA) erhalten [18, 34, 35]. Darüber hinaus wurden zwischenzeitlich zahlreiche Real-world-Studien, Übersichtsarbeiten, einzelne prospektive sowie eine randomisiert kontrollierte Studie veröffentlicht, welche die Sicherheit, Verbesserung der glykämischen Kontrolle und Lebensqualität einzelner Open-source-AID demonstrierten und Vergleichbarkeit mit kommerziellen AID-Systemen zeigten [9, 26, 36–42].

Die kanadische Diabetesgesellschaft hat rezent einen Leitfaden für medizinisches Fachpersonal sowie ein Positionspapier publiziert [43, 44]. Die primär ableitbare Kernaussage ist, dass Kliniker:innen Menschen mit Typ-1-Diabetes mellitus und deren Betreuungspersonen unabhängig von der gewählten Diabetestechnologie unterstützen sollen. Der gemeinsame Entscheidungsprozess („shared decision-making“), aber auch die Autonomie von Menschen mit Diabetes mellitus müssen in den Vordergrund gestellt werden.

**Abb. 2:**
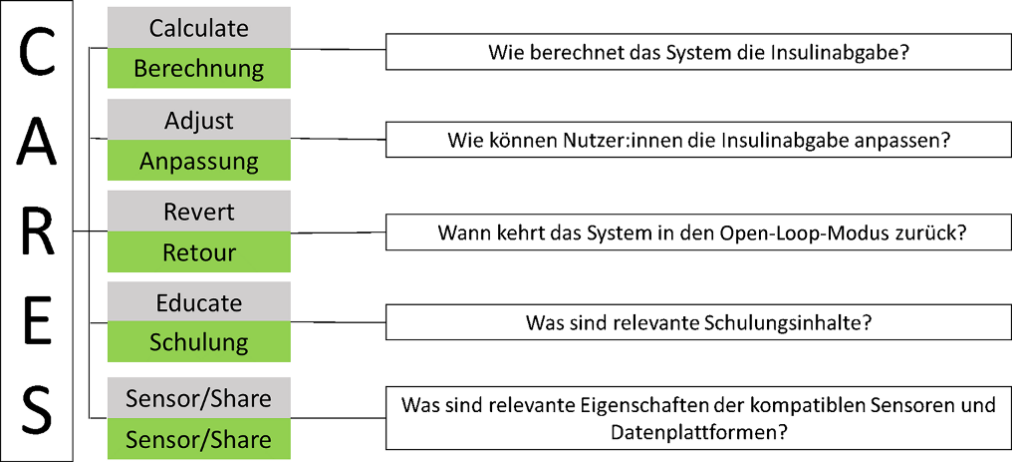
CARES-Modell. (Mod. nach [45])

Vonseiten des medizinischen Fachpersonals ist – wie allgemein für den Einsatz von Diabetestechnologie – ein Grundwissen notwendig, um Menschen mit Diabetes mellitus und deren Betreuungspersonen unterstützen zu können. Der Ansatz des CARES-Modells wird auch für die OS-AID-Betreuung empfohlen (Abb. 2; [43, 45]). Zur Recherche und vertiefenden Fortbildung für medizinisches Fachpersonal gibt es im Internet frei verfügbare Beiträge, Übersichtsartikel sowie die einschlägigen Webseiten einzelner Open-source-AID mit gezielter Information für klinisch tätiges Personal [29, 46–50]. In den aktuellen Leitlinien der Amerikanischen Diabetes Gesellschaft (ADA) wird hervorgehoben, dass Daten zu Sicherheit und Wirksamkeit einzelner Open-source-AID publiziert wurden [5].

## Open-source-Systeme – Überblick und Praxistipps

Wenn sich ein Mensch mit Diabetes mellitus für ein Open-source-AID entscheidet, sind ausreichend Vorarbeit und zeitliche Ressourcen notwendig. Hierbei muss recherchiert werden, welches Open-source-AID mit welcher Insulinpumpe, welchem CGM-System und welchem Smartphone kompatibel ist und welche Anforderung ein Computer erfüllen muss, um die mobile Applikation programmieren zu können [43]. All diese Informationen sind online frei verfügbar.

Derzeit existieren 4 Open-source-AID: IOS-Loop, AndroidAPS, OpenAPS und iAPS (vormals freeAPSX) [48, 51–54]. Der bereits angeführte Tidepool-Loop ist eine kostenpflichtige, downloadbare App, die auf dem IOS-Loop-Algorithmus basiert und als interoperable automatisierte glykämische Steuerung (iAGC) mit alternativ steuerungsfähigen Insulinpumpen (ACE-Pumpe) und integrierbaren CGM-Systemen (iCGM) via Bluetooth arbeitet mit dem Ziel, alle in der Garantiezeit befindlichen Insulinpumpen und CGM-Systeme herstellerunabhängig als AID-System verwenden zu können [35]. Die Tab. 1 erklärt relevante Begriffe bei Open-source-AID.

**Tab. 1:** Wichtige Begrifflichkeiten zu Open-source-AID [50]

Begriff (Abkürzung)	Begriffserklärung
Automated Insulin Delivery (AID)	Automatisiertes Insulinabgabesystem
Artificial Pancreas (AP)	Artifizielle/künstliche Bauchspeicheldrüse
Artificial Pancreas System (APS)	Künstliches Bauchspeicheldrüsensystem, Begriff wurde früher als Synonym zu HCL/AID verwendet; keine der verfügbaren AID-Optionen ist ein absolut selbstlaufendes System → Mahlzeiten müssen eingegeben werden; Hypoglykämiebehandlung durch Nutzer:in; daher unscharfe Formulierung
AndroidAPS (AAPS)	Android-App, Open-source-Implementierung eines APS mit einem Android-Smartphone, einer Version des OpenAPS-Algorithmus und einer Bluetooth-fähigen Pumpe
Autosens	(Automatische) Ermittlung und Änderung der Insulinempfindlichkeit (Bewegung, Hormone, Krankheit) unabhängig von Mahlzeiteneinfluss
Autotune	Automatische Kalkulation und je nach System auch Anpassung von ISF, CR und BR
Basal Rate (BR)	Basalrate wird in der Insulinpumpentherapie über 24 h programmiert
Carb Ratio (CR)	Kohlenhydratfaktor oder Kohlenhydrat:Insulin-Verhältnis sagt aus, wie viele Gramm Kohlenhydrate pro Einheit Insulin gegessen werden können
Carbs on Board (COB)	Nach einer Mahlzeit/einem Snack noch auf den Glukosespiegel einwirkende Kohlenhydrate
Do-it-Yourself (DIY)	Beschreibt die aktive Rolle, die Benutzer:in beim Programmieren und Updaten des eigenen AID-Systems auf Grundlage eines Open-source-Codes und der zugehörigen Programme einnehmen muss
Duration of Insulin Acting (DIA)	Im Körper aktiv wirksames Insulin, wird durch die Insulinwirkzeit vorgegeben
GitHub	Webbasierte Ablage für Entwickler, die dort Codes speichern, überarbeiten und teilen können
Hybrid-Closed-Loop(HCL)-System	Diabetesmanagementsystem, bestehend aus einem Glukosesensor, einer Insulinpumpe und einem Algorithmus, der anhand von Glukosewerten und Trends die Insulinabgabe automatisch anpassen kann
Insulin on Board (IOB)	Aktives Insulin im Körper, kann in Relation zur programmierten Basalrate negatives IOB oder positives IOB sein
Insulin-Sensitivitäts-Faktor (ISF)	Beschreibt, um wie viel die Blutglukose durch eine Einheit Insulin gesenkt wird
Loop	IOS-App, Open-source-Implementierung eines APS mit einem Apple IPhone, einem Open-source-Algorithmus, einer Insulinpumpe und einem CGM-System
MiaoMiao	Open-source-Transmitter, der aus Flash-CGM ein rt-CGM macht
Nightscout = „CGM in the Cloud“	Open-source-Datenverarbeitungstool für Open-source-AID-Systeme
OpenAPS	Erstes Open-source-AID-System mit kleinem Computer „rig“ zur Kommunikation mit Pumpe und CGM
Open-Source	Beschreibt die freie Veröffentlichung und gemeinsame Nutzung des Codes und der Algorithmen, die von der WeAreNotWaiting-Gemeinschaft ständig weiterentwickelt und verbessert werden
Real-time Continuous Glucose Monitoring (rt-CGM)	Kontinuierliches Glukosemesssystem, das automatisch Glukosewerte (alle 1–5 min) überträgt
Intermittently scanned Continuous Glucose Monitoring (is-CGM), auch Flash Glucose Monitoring (FGM)	Glukosemesssystem, bei welchem ein Scannen des Glukosesensors nötig ist, um aktuelle Glukosewerte zu erhalten
Super Micro Bolus (SMB)	Kleine Korrekturbolusgaben anstelle von temporärer Basalratenerhöhung
Tidepool	1. Datencloud (ähnlich Nightscout) 2. Tidepool Loop: erstes Open-source-AID mit FDA-Zulassung
Unannounced Meals (UAM)	Nicht angekündigte Mahlzeiten; Erkennung von signifikanten Erhöhungen des Glukosespiegels aufgrund von Mahlzeiten, Adrenalin oder anderen Einflüssen und Versuch, diese mit SMB anzupassen
#WeAreNotWaiting-Community	Gemeinschaft von Individuen, die Open-source-AID entwickelt haben, weil sie nicht auf kommerzielle Systeme warten wollten und die Belastung, mit Typ-1-Diabetes zu leben, frühzeitig überwinden wollten
xDrip+	Für Android, stellt CGM-Daten für AndroidAPS zur Verfügung; Alarme modifizierbar, Follow Apps

Im folgenden Teil sollen praktisch relevante Hinweise gegeben werden, die bei der Betreuung von Open-source-AID unterstützen können:

### Open-Loop-Modus

Beim Open-Loop-Modus berechnet der Algorithmus aus den verfügbaren Daten einen Behandlungsvorschlag, wie die Therapie angepasst werden soll. Die Vorschläge können manuell bestätigt werden. Die Verwendung des Open-Loop-Modus kann Menschen mit Diabetes mellitus, die sich für die Open-source-AID-Nutzung entscheiden, anfangs ermöglichen, das jeweilige System kennenzulernen und Vertrauen zu fassen. Bei AndroidAPS ist die anfängliche Nutzung des Open-Loop-Modus unumgänglich, und erst nach erfolgreicher Lösung verschiedener Aufgaben ist das Freischalten der Automation möglich [43, 52].

### Überprüfung der glykämischen Kontrolle

Bei Nutzung von Open-source-Technologie kann die glykämische Kontrolle mittels ambulantem Glukoseprofil (AGP) über das jeweilige CGM-Auswertungsportal überprüft werden. Alternativ ist eine detaillierte Datenauswertung über „Nightscout“ möglich. Es gelten auch bei Open-source-AID-Nutzung für die meisten Menschen mit Diabetes mellitus Typ 1 die international empfohlenen Ziele: Zeit im Zielbereich 70–180 mg/dl > 70 %, Zeit unter dem Zielbereich < 70 mg/dl < 4 % bzw. < 54 mg/dl < 1 % und Zeit über dem Zielbereich > 180 mg/dl < 25 % bzw. > 250 mg/dl < 5 % [55].

### Überprüfung der Basalrate

Eine passend programmierte und hinterlegte Basalrate ist essenziell für einen sicheren Notfallplan. Menschen mit Diabetes mellitus haben bei Open-source-AID-Nutzung kein klassisches Verhältnis zwischen Basal- und Bolusinsulin. In der Praxis können anhand des Vergleichs zwischen programmierter und tatsächlich abgegebener Basalrate – ohne Einfluss von Mahlzeiten oder Sport – Rückschlüsse gezogen werden. Hierbei kann eine häufig erhöhte Basalrate („positives Insulin On Board“ [IOB]) für ein zu schwach hinterlegtes Basalratenprofil sprechen, während umgekehrt kontinuierlich erniedrigte Basalraten („negatives IOB“) – v. a. in Kombination mit Hypoglykämien – zu starke Basalraten widerspiegeln können [43, 48, 51].

### Überprüfung der Boluseinstellungen

Der Insulinsensitivitätsfaktor (ISF) oder Korrekturfaktor gibt jene Menge an, um die eine Einheit Insulin den Glukosespiegel senken kann. Ein zu schwacher ISF führt hierbei zu Werten über dem Zielbereich, da Korrekturbolus und Basalanpassung zu schwach ausfallen würden. Umgekehrt führt ein zu aggressiver ISF zu Hypoglykämien. Nicht passende ISF-Einstellungen führen – in Abwesenheit von Mahlzeiteneinfluss – zu stark schwankenden Glukosewerten mit entsprechendem Tagesverlauf [43, 48].

### Mahlzeitenmanagement

Alle verfügbaren Open-source-AID sind Hybrid-Closed-Loop-Systeme, die die beste glykämische Kontrolle erzielen, wenn Mahlzeiten rechtzeitig und korrekt eingegeben werden. Je nach System und Algorithmus mit unterschiedlichen Parametern ist ein gewisses Ausgleichen von nicht eingegebenen Mahlzeiten („unannounced meals“ [UAM]) möglich. Vor Mahlzeiten kann ein präprandial niedrigerer Zielbereich als unter Normalbedingungen eingestellt werden. Dies kann helfen, postprandiale Hyperglykämien zu minimeren. Für die Behandlung einer Hypoglykämie werden in der Regel weniger Gramm Kohlenhydrate benötigt als bei sensorunterstützter Pumpen- oder multipler täglicher Injektionstherapie. Die konsumierten Hypoglykämiekohlenhydrate müssen nicht als Mahlzeit eingegeben werden, solange keine inadäquat hohe Kohlenhydratzufuhr erfolgt. Je nach System kann auch die Kohlenhydratabsorptionsrate angepasst werden, wenn eine Mahlzeit einen besonders hohen Protein- oder Fettanteil enthält [26, 51–53].

### Sicherheitseinstellungen und -empfehlungen

Bei Open-source-AID sind viele Einstellungen individualisierter vornehmbar, weshalb Sicherheitscharakteristika adäquat vorgegeben werden müssen. Die maximale Basalrate soll sinnvollerweise über der höchsten programmierten Basalrate liegen, da sonst eine weitere automatisierte Anpassung nach oben nicht möglich ist. Der maximale Bolus kann durch eine einzelne Dosis ohne Bestätigung vom System abgegeben werden und soll daher einen normalen – für das Individuum gängigen – Mahlzeitenbolus nicht überschreiten. Die Unterbrechungsschwelle („suspend threshold“) ist jener Glukosewert (durch den Algorithmus vorhergesagt oder aktuell), der eine weitere Insulinabgabe unterbricht. Wie auch bei kommerziellen AID-Systemen ist ein Notfallplan für den Fall eines technischen Versagens, bei Krankheit oder Reisen relevant und sollte jedem Menschen mit Diabetes mellitus zur Verfügung stehen. Wenn ein CGM-Signalverlust oder eine Störung der Kommunikation mit der Pumpe auftritt, kehrt das Open-source-AID nach einer definierten Zeit in den Open-Loop-Modus und greift auf die hinterlegte Basalrate zurück. So ist eine kontinuierliche Insulinzufuhr gewährleistet und das Risiko für das Auftreten einer diabetischen Ketoazidose minimiert. Da im Open-Loop-Modus keinerlei automatisierte Insulindosisanpassung (weder bei Hypo- noch bei Hyperglykämie) erfolgt, sind engmaschige Glukosekontrollen empfehlenswert. Auch für den Fall von ungenauen CGM-Werten empfiehlt es sich, bis zur Etablierung eines neuen Sensors in den Open-Loop-Modus zurückzukehren [26, 43, 45].

### Datenauswertung

Neben den oben genannten Open-source-AID gibt es für die zusammengeführte Datengenerierung und -auswertung von CGM und Insulinabgabe das ebenfalls aus der #WeAreNotWaiting-Gemeinschaft entwickelte – und nicht zugelassene – Tool „Nightscout“. Nutzer:innen können für die Visualisierung ihrer Daten eine individualisierte Web-URL mit dem medizinischen Personal teilen, über die die jeweiligen Daten angesehen und interpretiert werden können [43, 56, 57]. Die gemeinsame Beurteilung zusammengeführter Daten über Insulinzufuhr und CGM-Profile ist ein unbedingtes Erfordernis, um unterstützend als Behandler:in mitzuwirken.

## Mögliche Problemfelder und Lösungsansätze für die Praxis

Die größte Hürde im Bereich der Open-source-Technologie ist die – zum Großteil – fehlende Zulassung zur Nutzung als AID-System. Daher wird in der #WeAreNotWaiting-Gemeinschaft auch auf die Verwendung und Programmierung in Eigenverantwortung hingewiesen [51–53, 58]. Als medizinisches Fachpersonal ist die Verordnung zugelassener Einzelkomponenten jedenfalls gestattet, um Menschen mit Diabetes mellitus bei entsprechender Indikation Zugang zur Diabetestechnologie zu ermöglichen [26]. Menschen mit Diabetes mellitus sollen weiterhin im Diabetes-Management unterstützt werden, um im Ernstfall Sicherheit gewährleisten zu können. Auch Open-source-AID-Algorithmen basieren – je nach verwendetem System zumindest initial – auf hinterlegten Pumpeneinstellungen (Basalrate, Kohlenhydrat-zu-Insulin-Verhältnis, Insulinsensitivitätsfaktor), welche angepasst an den Insulinbedarf regelmäßig evaluiert werden sollen [5]. Hierbei ist es bei Kindern und Jugendlichen, bedingt durch das Wachstum bzw. die Gewichtszunahme und hormonelle Einflüsse in der Pubertät, relevant, den Insulinbedarf öfter (alle 3 bis 4 Monate) zu reevaluieren [59]. Wie bei kommerziell verfügbaren AID-Systemen muss jederzeit für mögliche Technologieversagen ein Notfallplan vorliegen. Ein Umstieg auf eine Basis-Bolus-Therapie mittels Pen muss gewährleistet sein und hierfür ausreichend Material zur Verfügung stehen. Eine regelmäßige Evaluierung der hinterlegten Pumpeneinstellungen des Open-Loop-Modus, also der Verwendung des Systems ohne automatisierte Steuerung, ist ebenso relevant.

Aus juristischem wie auch medizinischem Blickwinkel ist die Aufklärung über die fehlende Zulassung sowie die Verwendung auf Eigenverantwortung der Open-source-Technologien unbedingt notwendig. Relevant ist jedenfalls, dass das Programmieren von Open-source-Technologie selbstverantwortlich durch das Individuum mit Diabetes mellitus bzw. die Betreuungsperson erfolgen soll und hierfür nicht das medizinische Behandlungspersonal zuständig oder verantwortlich ist [44].

Hinsichtlich medizinisch-juristischer Bedenken wird im internationalen Konsensuspapier folgendes Vorgehen empfohlen: Im Sinne einer Diskussion sollen alle verfügbaren AID-Optionen mit Vor- und Nachteilen vorgestellt und die fehlende Zulassung von Open-source-Systemen soll erklärt werden. Die individuelle Entscheidung einer Person mit Diabetes mellitus bzw. deren Betreuungsperson ist zu respektieren, und weiterführende Unterstützung vonseiten des ärztlichen Personals soll gewährleistet werden, indem die zugelassenen Komponenten (Insulinpumpen, CGM-Systeme) weiterverordnet werden. Die Dokumentation soll klar und verständlich sein und bestätigen, dass das Individuum bzw. die Betreuungsperson sich der fehlenden Zulassung bewusst ist und weder das ärztliche Personal noch die behandelnde Klinik hierfür Verantwortung übernehmen kann. Eventuelle unerwünschte Ereignisse sollen – genauso wie bei kommerziellen AID-Systemen – rückgemeldet werden [26]. Im Sinne einer erfolgreichen und sicheren Anwendung sollen, auch bei Entscheidung für Open-source-Nutzung, eine strukturierte Schulung und grundlegende Kenntnisse zum Diabetes-Selbstmanagement (Reaktion auf Hyper- und Hypoglykämie, Symptomerkennung und Vorgehen bei Verdacht auf diabetische Ketoazidose, besondere Situationen wie Reisen, Sport und Krankheit) und Grundlagen der Insulinpumpentherapie und CGM-Nutzung vermittelt, überprüft und ggf. aufgefrischt werden. Relevant ist, dass für jedes Open-source-AID unterschiedliche Parameter relevant sind, Anforderungen bestehen und – möglicherweise dynamischer als bei kommerziellen AID-Systemen – mit regelmäßigen Weiterentwicklungen zu rechnen ist. Eine kontinuierliche Auseinandersetzung mit eventuellen Updates (sog. „branches“) ist somit erforderlich und obliegt der Eigenverantwortung der Anwender:innen. Im Sinne einer gemeinsamen Entscheidungsfindung zwischen Menschen mit Diabetes mellitus bzw. deren Betreuungsperson und medizinischem Fachpersonal dürfen Therapieziele unabhängig von der Nutzung von Open-source-Technologie definiert und reevaluiert werden, insbesondere auch in Bezug auf Screening, Verlaufskontrolle und Therapie von diabetesassoziierten Komplikationen und relevanten Komorbiditäten.

Menschen mit Diabetes mellitus bzw. deren Angehörige müssen trotz verfügbarer Therapieoptionen mit technologischer Unterstützung tagtäglich Echtzeit-Therapieentscheidungen treffen, die gewisse Risiken mit sich bringen. Gerade deswegen sollte die Autonomie von Menschen, die mit Diabetes mellitus leben, im Vordergrund stehen, um die individuell am besten geeignete Therapiemodalität zu finden.

Es gelten die Grundprinzipien der biomedizinischen Ethik – Autonomie, Wohltun, Nichtschaden und Gerechtigkeit. Im Sinne der Autonomie bei Open-source-AID-Nutzung ist relevant, dass Menschen mit Diabetes mellitus, die sich für diese Therapie entscheiden, potenzielle Risiken und Vorteile der Systeme verstehen. Passend dazu ist hervorzuheben, dass Open-source-Technologie Transparenz der Algorithmusfunktionsweise und Individualisierung der Einstellungen erlaubt, die bei kommerziellen AID-Systemen und -Algorithmen mitunter nur eingeschränkter gewährleistet werden können. Aspekte der Gerechtigkeit lassen sich durch den freieren globalen Zugang und die Kompatibilität zwischen Insulinpumpen und CGM-Systemen unterschiedlicher Herstellerfirmen erkennen. Die Prinzipien des Nichtschadens und Wohltuns lassen sich aus oben angeführten Real-world- und Observationsstudien ableiten, in denen Open-source-Systeme die Verbesserung der Zeit im Zielbereich und Minimierung von Hypoglykämien demonstrierten [26, 44, 60]. Auch wenn Open-source-Algorithmen im Internet frei abrufbar sind, um alle Menschen mit Diabetes mellitus zu erreichen, zeigt sich im Real-world-Setting, dass diese v. a. von Menschen mit höherem Bildungsgrad, höherem Einkommen, technischen Fähigkeiten und Kenntnissen in Informatik verwendet werden [22, 61]. Es wird eine Diskussion aller verfügbaren Behandlungsoptionen mit vorliegender Evidenzlage hinsichtlich Vor- und Nachteile empfohlen, um einen informierten Entscheidungsprozess der Menschen mit Diabetes mellitus zu ermöglichen.

Für alle – das Betreuungsteam und Menschen mit Diabetes mellitus, die Open-source-Technologie betreuen oder nutzen – sind die raschen Entwicklungsfortschritte in diesem Bereich herausfordernd. Das Lernen von- und miteinander ist Teil eines Betreuungskonzepts auf Augenhöhe [62]. Bei fehlender Expertise mit AID-Systemen empfiehlt sich eine Zuweisung an ein Zentrum mit entsprechender Erfahrung [43]. Jedenfalls existieren, wie oben angeführt, Handlungsempfehlungen, Konsensuspapiere sowie Evidenz für Open-source-AID und Leitfäden für medizinisches Fachpersonal, was synergistisch wirkend zu einem großen Wissenszuwachs führen kann [26, 37, 43, 44, 47, 50].

## Konklusion

Anhand vorliegender Evidenz kann abgeleitet werden, dass mit Open-source-AID die glykämische Kontrolle und Lebensqualität verbessert werden können und dass die Systeme bei korrekter Nutzung sicher sind. Entsprechend den Grundsätzen medizinischer Ethik sollen alle verfügbaren Therapieoptionen für die Insulintherapie diskutiert und hierbei Risiken kritisch besprochen werden, damit eine informierte und individuelle Entscheidungsfindung gewährleistet werden kann. Menschen mit Diabetes mellitus und deren Betreuungspersonen, die sich eigenverantwortlich für die Nutzung von Open-source-Technologie entscheiden, müssen weiterhin durch ärztliches Personal betreut und unterstützt werden. Unabhängig vom gewählten AID-System sind eine strukturierte Schulung, grundlegende Kenntnisse im Diabetes-Selbstmanagement (Reaktion auf Hyper- und Hypoglykämie, Symptomerkennung und Vorgehen bei Verdacht auf diabetische Ketoazidose, besondere Situationen wie Reisen, Sport und Krankheit) gemeinsam mit Grundlagen der Insulinpumpentherapie und eine kontinuierliche Betreuung notwendig, um eine sichere und zielgerichtete Therapie der metabolischen Einstellung, akuter und chronischer Komplikationen sowie das Screening auf assoziierte Erkrankungen zu gewährleisten. Diese soll als multidisziplinäres Team mit entsprechender Expertise erfolgen.

## Open-source-Technologie – Empfehlungen für die Praxis

- Menschen mit Diabetes mellitus bzw. deren Familie, Eltern, Erziehungsberechtigte oder gesetzliche Vertreter:innen verwenden Open-source-Technologie eigenverantwortlich.
- Menschen mit Diabetes mellitus und deren Betreuungspersonen sollen über die – zum Großteil – fehlende Zulassung von Open-source-AID aufgeklärt werden, und Risiken müssen diskutiert werden.
- Das Programmieren und Installieren von Open-source-AID sollen durch Menschen mit Diabetes mellitus bzw. deren Betreuungsperson selbst erfolgen, eine Peer-to-peer-Unterstützung aus der Gemeinschaft ist möglich. Dadurch werden ein Auseinandersetzen mit der Technologie, der Community und mögliche Fehlerbehebungen gewährleistet.
- Ärzt:innen und Diabetesberater:innen sollen über alle verfügbaren Therapieoptionen für Insulintherapie, inklusive kommerzieller AID-Systeme und Open-source-AID, informieren.
- Zugelassene Einzelkomponenten (CGM/Insulinpumpe) können durch das behandelnde Diabetesteam verordnet werden.
- Eine ärztliche Weiterbetreuung und Unterstützung im Diabetes-Management zum Erreichen der individuellen Therapieziele sind möglich, sinnvoll und erforderlich.
- Open-source-AID soll nicht bevorzugt zu kommerziell zugelassenen AID-Systemen empfohlen werden. Die beste Lösung für jeden einzelnen Menschen mit Diabetes mellitus soll individuell gesucht und gegenüber potenziellen Risiken abgewogen werden.
- Menschen mit Diabetes mellitus sollen weiterhin im Diabetes-Management unterstützt werden, um Sicherheit zu gewährleisten. Wie bei kommerziell verfügbaren und zugelassenen AID-Systemen soll ein Notfallplan für den Fall eines Pumpenversagens vorliegen.
- Die kontinuierliche Langzeitbetreuung von Menschen mit Diabetes mellitus mit Open-source-AID soll durch multidisziplinäre Teams mit entsprechender Expertise erfolgen.

## Abkürzungen

AAPS: AndroidAPS
ACE-Pumpe: Alternativ steuerungsfähige Insulinpumpe
ADA: Amerikanische Diabetes Gesellschaft
AGP: Ambulantes Glukoseprofil
AID-Systeme: Automatisierte Insulinabgabesysteme
AP: Artificial Pancreas
APS: Artificial Pancreas System
BR: Basal Rate
CGM: Kontinuierliches Glukosemesssystem
COB: Carbs on Board
CR: Carb Ratio
CSII: Kontinuierliche subkutane Insulinzufuhr; Insulinpumpe
DIA: Duration of Insulin Acting
DIY: Do-it-Yourself
DIYPS: Do-it-Yourself-Pancreassystems
fCGM: Flash CGM
FDA: Food and Drug Administration
HCL-Systeme: Hybrid-Closed-Loop-Systeme
HCP: Health-care Professional
iAGC: Interoperable automatisierte glykämische Steuerung
iCGM: Integrierbare CGM-Systeme
IOB: Insulin on Board
is-CGM: Intermittently scanned CGM
ISF: Insulin-Sensitivitäts-Faktor
MDI: Multiple tägliche Injektionstherapie
Open-source-AID: Open-source-AID-Systeme
rt-CGM: Real-time CGM
TIR: Zeit im Zielbereich
UAM: Unannounced meals = nicht eingegebene Mahlzeit

## Interessenkonflikt

A.-T. Kietaibl, L. Frühwald, M. Resl, M. Eichner und H. Stingl geben an, dass kein Interessenkonflikt besteht. B. Rami-Merhar: Advisory boards or speakers honorary in the last 3 years: Abbott, Medtronic, Sanofi, Eli Lilly, Insulet, Dexcom, Novo Nordisk, Ypsomed. C. Schelkshorn hat von folgenden Unternehmen Forschungsunterstützungen und/oder Honorare erhalten: Novo Nordisk, Lilly Austria, Böhringer Ingelheim, AstraZeneca, Abbott Diabetes, Sanofi, Daiichi Sankyo. E. Fröhlich-Reiterer hat in den letzten 36 Monaten von folgenden Firmen Vortragshonorare, Forschungsgelder und Honorare für Beratungstätigkeit erhalten: Medtronic, Eli Lilly, Sanofi und Merck, Sandoz. G. Köhler hat von folgenden Unternehmen Forschungsunterstützungen und/oder Honorare erhalten: Amgen, AstraZeneca, Boehringer Ingelheim, Eli Lilly, Novo Nordisk, Novartis, Roche Diagnostics, Sanofi Aventis, Viatris. G. Rega-Kaun hat von folgenden Unternehmen Kongressunterstützungen und/oder Honorare erhalten: Abbott, Boehringer Ingelheim, Eli Lilly, Novo Nordisk, Sanofi. I. Schütz-Fuhrmann hat von folgenden Unternehmen Honorare erhalten: Abbott, Med Trust, Medtronic, Dexcom. J.K. Mader ist in den Advisory Boards von Abbott Diabetes Care, Becton Dickinson/Embecta, Biomea Fusion, Eli Lilly, Medtronic, Novo Nordisk, Pharmasens, Roche Diabetes Care, Sanofi und Viatris. Sie erhielt Sprecherhonorare von Abbott Diabetes Care, A. Menarini Diagnostics, Becton-Dickinson/Embecta, Eli Lilly, Med Trust, Novo Nordisk, Roche Diabetes Care, Sanofi und Ypsomed. Sie ist Gesellschafterin von decide Clinical Software GmbH und elyte Diagnostics. L. Bozkurt hat in den letzten 36 Monaten von folgenden Firmen Honorare erhalten: Medtronic, Sanofi, Novo Nordisk. L. Stechemesser hat von folgenden Unternehmen Forschungsunterstützungen und/oder Honorare erhalten: Abbott, Amgen, AstraZeneca, Bayer, Boehringer Ingelheim, Dexcom, Eli Lilly, Medtronic, Med Trust, MSD, Mylan, Novartis, Novo Nordisk, Roche Diabetes Care, Sanofi Aventis. M. Riedl hat Unterstützungen der Firma Novo und Böhringer für Kongressteilnahme erhalten. M. Tauschmann hat in den letzten 36 Monaten von folgenden Firmen Vortragshonorare und Honorare für Beratungstätigkeit erhalten: Abbott, Eli Lilly, Medtronic, Sanofi und Ypsomed. R. Weitgasser hat von folgenden Unternehmen Forschungsunterstützungen und/oder Honorare erhalten: Abbott Diabetes Care, Eli Lilly, Medtronic, Med Trust, Novo Nordisk, Roche Diabetes Care. S.E. Hofer hat von folgenden Unternehmen Forschungsunterstützungen und/oder Honorare erhalten: Sanofi, Eli Lilly, Medtronic, Ypsomed, Insulet, Dexcom. S. Zlamal-Fortunat war als Vortragende für zahlreiche Pharmafirmen in den letzten 3 Jahren tätig: Med Trust, Medtronic, AstraZeneca, Novartis, Novo Nordisk, Eli Lilly, Böhringer Ingelheim, Daiichi Sankyo und Sanofi. T. Hörtenhuber hat von folgenden Unternehmen Forschungsunterstützungen und/oder Honorare erhalten: Medtronic, Sanofi, Merck, Lilly. Y. Winhofer hat von folgenden Unternehmen Forschungsunterstützungen und/oder Honorare erhalten: Abbott, Eli Lilly, Medtronic, Novo Nordisk, Sanofi Aventis.
